# What is central to renal nutrition: protein or sodium intake?

**DOI:** 10.1093/ckj/sfad151

**Published:** 2023-06-29

**Authors:** Angela Yee-Moon Wang, Francesca Mallamaci, Carmine Zoccali

**Affiliations:** University Department of Medicine, Queen Mary Hospital, The University of Hong Kong, Hong Kong, SAR, China; Nefrologia and CNR Unit, Grande Ospedale Metropolitano, Reggio Calabria, Italy; Renal Research Institute, New York, USA; Institute of Molecular Biology and Genetics (Biogem), Ariano Irpino, Italy; Associazione Ipertensione Nefrologia Trapianto Renal (IPNET), Nefrologia, Grande Ospedale Metropolitano, Reggio Calabria, Italy

**Keywords:** CKD, implementation, protein diet, renal nutrition, sodium restricted diet

## Abstract

Historically, nutrition intervention has been primarily focused on limiting kidney injury, reducing generation of uraemic metabolites, as well as maintaining nutrition status and preventing protein-energy wasting in patients with chronic kidney disease (CKD). This forms an important rationale for prescribing restricted protein diet and restricted salt diet in patients with CKD. However, evidence supporting a specific protein intake threshold or salt intake threshold remains far from compelling. Some international or national guidelines organizations have provided strong or ‘level 1’ recommendations for restricted protein diet and restricted salt diet in CKD. However, it is uncertain whether salt or protein restriction plays a more central role in renal nutrition management. A key challenge in successful implementation or wide acceptance of a restricted protein diet and a restricted salt diet is patients’ long-term dietary adherence. These challenges also explain the practical difficulties in conducting randomized trials that evaluate the impact of dietary therapy on patients’ outcomes. It is increasingly recognized that successful implementation of a restricted dietary prescription or nutrition intervention requires a highly personalized, holistic care approach with support and input from a dedicated multidisciplinary team that provides regular support, counselling and close monitoring of patients. With the advent of novel drug therapies for CKD management such as sodium-glucose cotransporter-2 inhibitors or non-steroidal mineralocorticoid receptor antagonist, it is uncertain whether restricted protein diet and restricted salt diet may still be necessary and have incremental benefits. Powered randomized controlled trials with novel design are clearly indicated to inform clinical practice on recommended dietary protein and salt intake threshold for CKD in this new era.

## INTRODUCTION

**Figure 1:**
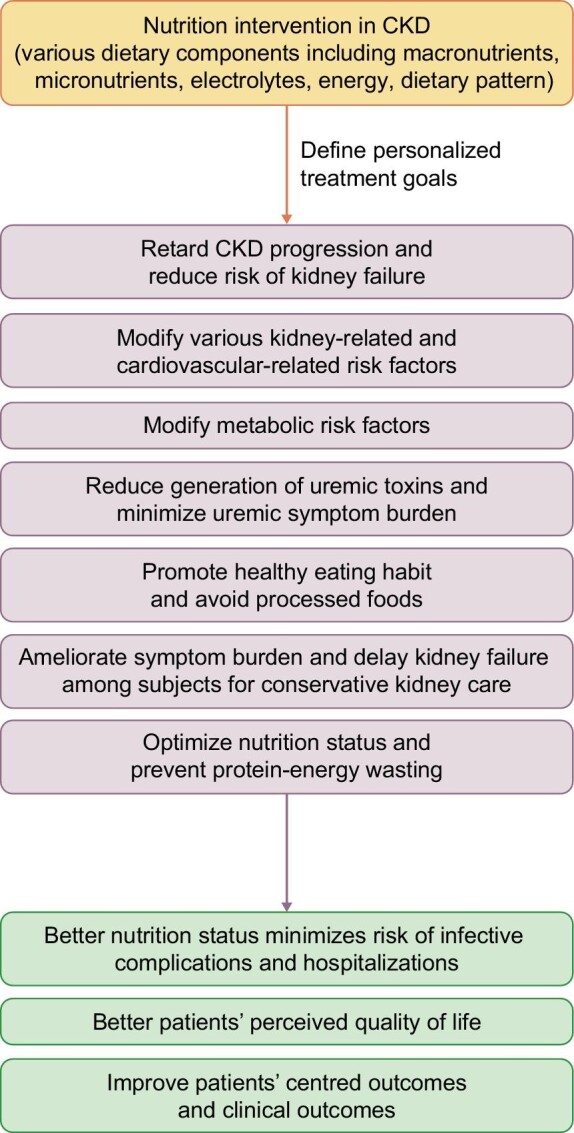
Goals of nutrition intervention in CKD.

Nutrition intervention is a cornerstone in chronic kidney disease (CKD) management and addresses multiple goals. Dietary modification instituted in CKD management aims to slow down CKD progression, delay the need for dialysis and lessen uraemic symptom burden as CKD progresses. It ameliorates the severity of cardiovascular-, metabolic- and kidney disease–related risk factors, reduces accumulation of uraemic metabolites and optimizes nutrition status of CKD subjects as their CKD progresses with eventual transition to kidney failure requiring dialysis [1]. Generally, subjects who maintained a better overall nutrition status had more favourable clinical outcomes, lower risk of infections, hospitalizations and mortality [2] (Fig. 1). Dietary protein intake of 0.8 g/kg/day is considered the minimum protein intake level required to maintain neutral nitrogen balance in stable patients without intercurrent illnesses and is generally recommended in early CKD stage 1–2 [3]. As CKD progresses to stage 3, a protein intake of 0.6–0.8/kg/day is usually prescribed [3]. A low protein diet (LPD) is defined as a dietary protein of 0.55–0.6 g/kg body weight/day while a very low protein diet (VLPD) as a dietary protein of 0.28–0.43 g/kg body weight/day [4]. Adequate energy intake of 30–35 kcal/kg/day is needed to avoid protein catabolism and protein-energy wasting for dietary protein intake ≤0.6 g/kg/day. Ketoanalogue supplementation is generally required with VLPD prescription to prevent nitrogen imbalance in CKD [4, 5]. The delivery of nutrition intervention requires specialized trained personnel to assess nutrition status and educate patients on various modifications required for different dietary components including protein, fat, energy, salt, potassium, phosphorus, and other vitamins and minerals [4]. There are also emerging data to support a healthy dietary pattern in CKD subjects [6]. However, randomized controlled trials that evaluated the impact of different elements of dietary modifications on hard outcomes were lacking in CKD population. Although the global clinical practice guidelines such as Kidney Disease: Improving Global Outcomes (KDIGO) and American Diabetes Association clinical practice guidelines made a strong recommendation on lifestyle modification in managing diabetes in CKD [7, 8], the evidences on which the statement was drawn were mostly based on observational data or data from the general population. In this paper, we review the rationale and the basis of the current dietary modifications recommended for patients with CKD with and without diabetes and discuss the relative importance of modifying protein and salt intake in improving outcomes of this population.

## MECHANISMS OF LOW PROTEIN DIET IN CHRONIC KIDNEY DISEASE

The observation of a liberalized or high protein intake causing adverse effects on the kidneys in experimental models [9] and in humans [10] dates back to the 1970s. Human volunteers fed a high red meat diet showed an acute increase in glomerular filtration rate (GFR) with a proportional increase in renal blood flow and reduction in renal vascular resistance as a result of afferent arteriolar dilatation [10]. High protein diet increased plasma renin activity and aldosterone level which was not reversed by chronic angiotensin-converting enzyme inhibition [11]. The adverse haemodynamic and neurohormonal effects observed with a high protein diet translated to more glomerulosclerosis and proteinuria over time in both experimental models with intact or reduced renal mass compared with those being fed an LPD [12]. A high protein diet induced proinflammatory gene expression, renal lipogenesis, initiated glomerular hypertension and hyperfiltration, leading to progressive glomerulosclerosis in experimental models of kidney disease [13–15]. On the other hand, dietary protein restriction retarded glomerular injury and glomerulosclerosis by constricting afferent arterioles, thus ameliorating glomerular hyperfiltration and hypertension [12]. LPD also repressed inflammation and oxidative stress through upregulation of Kruppel-like factor-15, a negative regulator of fibrosis in mesangial cells, limiting glomerulosclerosis [16]. Notably, restricted dietary protein preserves kidney function and limits structural injury even when instituted in the setting of established kidney injury [17]. These evidences form the scientific basis of prescribing restricted protein diet in kidney diseases [18, 19]. In an experimental model, combined treatment of LPD with angiotensin receptor blockade was more effective in lowering proteinuria, transforming growth factor-β overexpression and glomerular matrix accumulation than treatment with renin–angiotensin system blockade alone [20].

Box 1: Experimental data showed that dietary protein restriction may ameliorate glomerular hyperfiltration, glomerular hypertension and glomerulosclerosis by afferent arteriolar constriction. Dietary protein restriction also limits inflammation and oxidative stress and upregulates Kruppel-like factor-15, thus limiting glomerulosclerosis.

Combined treatment of restricted protein diet and angiotensin receptor blockade may be more effective than angiotensin receptor blockade alone in lowering proteinuria and ameliorating glomerular injury and glomerulosclerosis in experimental models.

## CLINICAL STUDIES WITH LOW PROTEIN DIET

The experimental evidences were supported by observational studies suggesting an association between higher dietary protein intake and more rapid decline in kidney function among subjects with and without CKD [21–24]. Higher protein intake was reported to be associated with more rapid decline in kidney function post-myocardial infarction [25]. A small study suggested an additional antiproteinuric effect by combining LPD with angiotensin-converting enzyme inhibitor treatment in proteinuric kidney disease [26]. However, data from randomized controlled trials (RCTs) evaluating restricted protein diet in humans have yielded mixed data. In the Modification of Diet in Renal Disease (MDRD) study that recruited non-diabetic CKD stage 2–3 subjects with a mean GFR 38 mL/min/1.73 m^2^, no difference was observed in measured GFR decline and kidney failure among subjects randomized to LPD versus those to usual protein diet over a 3-year follow-up. In CKD stage 4–5 subjects with a mean GFR 19 mL/min/1.73 m^2^, those randomized to a VLPD (0.28 g/kg/day) plus essential amino acid and keto-acid supplement tended to have less GFR decline than those randomized to LPD, but there was no effect on kidney failure [27]. The primary results of the MDRD study were considered negative. However, in the secondary analysis of MDRD study, the analysis was stratified into two phases. In the initial 4 weeks, a more rapid decline in measured GFR was observed with LPD compared with usual protein diet with a mean difference of 1.6 mL/min/1.73 m^2^ per year. Subsequent to the first 4 weeks, subjects receiving LPD showed a significantly lower decline in measured GFR compared with those receiving usual protein diet with a mean difference of 1.1 mL/min/1.73 m^2^ per year, suggesting a potential kidney protective benefit of LPD [28]. The initial dip or fall in GFR observed with LPD before GFR stabilized was attributed to reversible glomerular haemodynamic changes. In another secondary analysis of the MDRD study that combined patients assigned to both diets, having a 0.2 g/kg/day lower achieved total protein intake (including food and supplement) was associated with a 1.15 mL/min/1.73 m^2^ per year slower mean decline in GFR (*P* = .011), equivalent to a 29% reduction in mean GFR decline. This was translated to a 41% prolongation in the time to kidney failure in subjects with advanced kidney disease [29]. However, in a long-term follow-up of MDRD Study, assignment to a VLPD did not delay progression to kidney failure, but appeared to increase the risk of death compared with the group assigned to LPD [30]. Several other studies that evaluated the efficacy of LPD on rate of kidney function decline or kidney failure outcomes yielded rather mixed results and the studies were very dated [31–34]. A more contemporary prospective RCT conducted in non-diabetic CKD patients with estimated GFR (eGFR) <30 mL/min/1.73 m^2^ demonstrated efficacy of a vegetarian VLPD supplemented with ketoanalogues in lowering the risk of reaching primary composite endpoint of kidney replacement therapy or a 50% reduction in the initial GFR in both the intention to treat (ITT) analysis and per protocol (PP) analysis over 15 months of treatment. The adjusted numbers needed to treat to avoid the primary composite endpoint in both ITT and PP analysis in one patient was 4.4 (4.2–5.1) and 4 (3.9–4.4), respectively, for subjects with eGFR <30 mL/min/1.73 m^2^. Adjusted numbers needed to treat to avoid dialysis was 22.4 (21.5–25.1) for subjects with eGFR <30 mL/min/1.73 m^2^ but decreased to only 2.7 (2.6–3.1) for subjects with eGFR <20 mL/min/1.73 m^2^ [35]. However, only 14% of screened patients were recruited into the study and randomized. The prescribed protein intake was very low, only 0.3 g/kg/day. This raises questions on the generalizability and wider applicability of the results and patients’ long-term adherence and compliance to such low levels of protein intake.

Over the past 20 years, several systematic reviews have evaluated RCTs of LPD or VLPD in CKD [36–38]. In the most recent systematic review by Hahn and co-workers [37], 17 RCTs or quasi-RCTs conducted in a total of 2996 subjects with non-diabetic CKD with a mean follow-up duration ranging from 12 to 50 months were pooled and analysed. The results showed that VLPD probably slowed the progression to end-stage kidney disease (ESKD) but the benefits of LPD were marginal with no benefits in reducing the risk of kidney failure. LPD or VLPD did not influence death. Based on these analysis, the Kidney Disease Outcome Quality Initiative (KDOQI) nutrition guidelines in CKD 2020 recommended a restricted protein diet in non-diabetic CKD, namely a diet providing 0.55–0.60 g dietary protein per kg ideal body weight per day, or a VLPD providing 0.28–0.43 g dietary protein per kg ideal body weight per day with additional keto acid analogues to meet protein requirements [4]. A recent economic analysis suggested that a ketoanalogue-supplemented VLPD significantly increases quality-adjusted life-years, lowers lifetime care cost, and is a cost-effective strategy in retarding CKD progression and postponing dialysis compared with a conventional LPD in patients with CKD 4–5 [39]. However, implementation of a restricted protein diet is not without challenge and requires a personalized approach with dietitians and multidisciplinary team input, as well as regular and close patient monitoring [40].

On the other hand, a few systematic reviews have attempted to examine LPD/VLPD in diabetic CKD but the studies included all had a very small sample size and were not designed to examine death or kidney failure. Thus, it remained inconclusive whether LPD/VLPD modifies diabetic CKD [41, 42]. On the basis of the very limited evidence, both KDIGO and American Diabetes Association recommended a dietary protein intake of 0.8 g/kg/day in diabetic CKD as in the general population [7]. The United Kingdom Kidney Association recommended a protein intake of 0.8–1.0 g/kg ideal body weight per day for patients with stage 4–5 CKD not on dialysis (1C) [43]. This protein intake level of 0.8–1.0 g/kg/day is not really restricted. A lower target for dietary protein intake of 0.6–0.8 g/kg body weight per day was suggested by KDOQI nutrition guidelines for diabetic CKD 3–5 to maintain a stable nutritional status and optimize glycemic control, and this was purely opinion based [4] (Table 1).

**Table 1:** Summary of various guidelines recommending dietary protein intake and salt intake level in CKD.

	KDOQI 2020	KDIGO BP 2021	KDIGO DM 2022	UKKA 2019	ESPEN 2021 [81]	KHA-CARI 2013 [82]
Protein	CKD 3–5 and DM: 0.6–0.8 g/kg/day (opinion)		DM and CKD ND: 0.8 g/kg/day (2C)	CKD 4–5: 0.8–1.0 g/kg/day	Hospitalized CKD patients without acute/critical illness: 0.6–0.8 g/kg/day	CKD early stage and stage 3–5: 0.75–1.0 g/kg/day with adequate energy
	CKD 3–5 and non-DM:					
	LPD: 0.55–0.60 g/kg/day or					
	VLPD: 0.28–0.43 g/kg/day with KA to meet protein requirements of 0.55–0.60 g/kg/day (1C to improve QOL) (1A to reduce risk of ESKD/death)					
						
Sodium	CKD 3–5, limit sodium intake to <100 mmol/day (or <2.3 g/day) to reduce BP and volume control (1B)	Patients with high BP and CKD, target a sodium intake <2 g of sodium per day (or <90 mmol of sodium per day, or <5 g sodium chloride per day) (2C)	Patients with DM and CKD, sodium intake target <2 g sodium per day (or <90 mmol of sodium per day, or <5 g of sodium chloride per day) (2C)	Propose reducing dietary salt intake to a maximum of 6 g/day for CKD with high BP		Restrict to <100 mmol sodium per day or 2.3 g/day sodium or 6 g salt per day
	CKD 3–5, limit sodium intake to <100 mmol/day (or <2.3 g/day) to reduce proteinuria synergistically with pharmacologic interventions (2A)					

BP, blood pressure; DM, diabetes mellitus; UKKA, United Kingdom Kidney Association; ESPEN, European Society or Parenteral and Enteral Nutrition; QOL, quality of life; ND, non-dialysis; KHA-CARI, Kidney Health Australia—Caring for Australasians with Renal Impairment; KA, Keto-analogue.

In a recent pragmatic RCT from Italy, 229 advanced CKD subjects were randomized to receive VLPD (0.35 g/kg/day) or LPD (0.6 g/kg/day). Patients were followed for a median of 74.2 months. The primary outcome was time to kidney death, defined as the first event between kidney failure and all-cause mortality; secondary outcome was cardiovascular events. The study showed no additional benefit of prescribing a VLPD on kidney or survival outcomes in patients with advanced CKD under regular nephrology care. Furthermore, adherence to protein restriction is low and poses a key practical barrier in widespread acceptance and implementation of LPD in CKD [44].

## DOES RESTRICTED PROTEIN DIET HAVE ANY BENEFITS BEYOND THE KIDNEYS?

Box 2: Recent systematic review pooling the randomized trials in the last 20 years or so showed that VLPD probably slowed the progression to ESKD but the benefits of LPD were marginal, with no benefits for kidney failure. LPD or VLPD did not influence death.

There are some hypothetical metabolic benefits that restricted protein diet may have beyond its effects on the kidneys. Protein and sodium are frequently associated in many foods. Thus, a restricted protein diet also lowers sodium intake and improves blood pressure control in subjects with advanced CKD [45]. Restricted protein diet may improve lipid and glucose control in diabetic kidney disease [42, 46]. It reduced phosphorus load and generation of fibroblast growth factor-23 (FGF-23) [47]. High phosphorus levels attenuated the anti-proteinuric effects of VLPD and angiotensin-converting enzyme inhibition in CKD [48, 49]. Restricted protein diet reduced generation of metabolic acids, urea nitrogen and various uraemic metabolites such as p-cresol sulfate and indoxyl sulfate. These uraemic metabolites may induce toxic effects on mesangial cells, tubular cells, cardiac myocytes and vascular endothelial cells, causing both cardiovascular disease and kidney injury [50]. Further evaluation and confirmation are required with RCTs.

## DOES THE QUALITY OF PROTEIN MATTER?

Emerging data from observational studies has suggested that the quality and source of protein may matter in CKD. There are some suggestions that plant protein may prevent an increase in FGF-23 [51] and reduce the degree of metabolic acidosis compared with animal protein in CKD [52]. Dietary animal protein intake leads to an imbalance of gut microbes with a relative abundance of proteolytic gut microbiota profile [53], resulting in generation of more gut-derived uraemic toxins including p-cresol, indoxyl sulfate and trimethyl-amine N-oxide (TMAO) which have adverse effects on cardiovascular and kidney axis [50, 54]. The increased generation of nitrogenous wastes was thought to contribute to uraemic symptoms burden among subjects with CKD. Increased circulating TMAO [55] has also been shown to increase risk of mortality as well as adverse cardiovascular and kidney outcomes [56, 57]. Higher intakes of animal protein may be associated with increased risk of type 2 diabetes while higher plant protein intake tended to be associated with lower risk of type 2 diabetes [58]. Plant proteins may lower the incidence of new-onset CKD in the general population cohort [59, 60]. In the Singapore Chinese Health Study of over 63 000 general population subjects, a strong dose-dependent relationship was observed between red meat intake and increased risk of kidney failure over 15.5 years of follow-up [61]. Replacing one serving of red meat intake with other food sources of protein was associated with a maximum relative risk reduction of kidney failure of 62% [61]. A recent female aging cohort also observed a slower decline in eGFR among subjects who consumed a diet richer in plant-sourced protein, namely fruits, vegetables and nut-derived protein, versus those who did not [62]. In line with these data from the general population, the National Health and Examination Survey (NHANES) III showed that adult subjects with eGFR <60 mL/min/1.73 m^2^ who consumed a diet with higher proportion of plant-sourced proteins was associated with lower all-cause mortality over an average follow up of 8.4 years [63]. On the other hand, consumption of more plant proteins may not be associated with higher risk of high serum potassium or phosphate levels, but rather with higher fiber intake and better diet quality [64, 65]. However, all these studies were either observational or retrospective in design. The overall quality of evidence is low and the clinical implications of these studies remain very uncertain. RCTs will be required to evaluate the difference between plant-based versus animal-based diet in relation to various clinical outcomes in patients with CKD. Of note, existing studies that examined restricted protein diet or quality of dietary protein were done before the era of sodium-glucose cotransporter-2 (SGLT-2) inhibitors or non-steroidal mineralocorticoid receptor antagonist (MRA). It remains to be evaluated whether there is an incremental benefit of a restricted protein diet additional to the guideline-directed medical therapies, namely renin–angiotensin–aldosterone system blockade and SGLT-2 inhibitors, in preventing CKD progression and kidney failure.

Box 3: Although there are theoretical metabolic benefits of a plant-based protein diet compared with an animal-based protein diet for CKD, the overall quality of evidence remain low and clinical implications of these studies remain uncertain.

## BARRIERS IN IMPLEMENTATION OF RESTRICTED PROTEIN DIET IN CHRONIC KIDNEY DISEASE

One potential reason for the negative findings in several of the trials and a key contributor to barriers in implementing LPD/VLPD in clinical practice relates to patients’ acceptance and adherence to LPD/VLPD, especially in the long-term. These barriers will only increase in the future, especially with the availability of more effective drugs in retarding CKD progression [66]. Adherence to a protein-restricted diet was reported be around 60%–70% and may even be lower with VLPD. Although patients’ acceptance of a VLPD may be no more than 20%, subjects may still benefit from a reduced protein load with generation of fewer uraemic toxins [35]. To improve implementation and adherence to an LPD/VLPD, a holistic multidisciplinary care approach providing regular dietary counselling and support, with close monitoring of patients, is essential [67]. However, the reality is that this approach is a chimera worldwide. Only selected centres with a prominent scientific interest or expertise on renal nutrition will be able to implement a ‘holistic personalized care approach’ in nutrition intervention. In many parts of the world and especially in low resources settings, there are significant shortages of dietitians/renal dietitians, as reported by the Global Kidney Nutrition Care Atlas [68]. This poses a huge challenge in the delivery of kidney nutrition care to the much-needed CKD population.

Box 4: There are practical barriers to the widespread application and implementation of a restricted protein diet in CKD patients. The significant global shortages of dietitians/renal dietitians/personnel with specialized training in renal nutrition especially in low resources settings pose an important barrier to the effective delivery of kidney nutrition care in CKD population.

## SODIUM RESTRICTION IN CHRONIC KIDNEY DISEASE

In the late 18th century, salt was not tainted with the stigma of causing water retention, oedema and kidney disease. The idea that salt restriction rather than salt excess may be noxious for human health prevailed in those years [69]. At the turn of the century, ideas on salt and human diseases changed radically. In 1901, Achard suggested that the oedema of Bright's disease (chronic inflammation of the kidneys) was caused by chloride retention and secondary water accumulation. He argued that salt retention was the cause, rather than the effect of several diseases. In 1904, Ambard and Beaujard tested the effect of a low-salt diet (3 g of salt, 1.2 g of sodium) compared with a high-salt diet (14 g of salt or 5.8 g of sodium) in six hypertensive patients, some of whom had Bright's disease. In this study they noted that ‘…the changes in blood pressure were not striking but tended to be downward when the low salt diet was given and upward when the higher salt intake was allowed’ [69]. Kempner recognized the benefits of avoiding excess salt intake in CKD patients. He invented the ‘rice diet’, a demanding dietary regimen restricting sodium intake to 0.15 g/day (virtually saltless diet) and noted that this diet substantially reduced oedema and other complications of fluid overload [69]. From the 1960s on, reduction of dietary salt gained momentum for the prevention and treatment of cardiovascular and kidney diseases. The Dietary Guidelines for Americans 2020–2025 recommend a 2.3 g sodium (5.8 g salt) diet in the general population [70] and the American Heart Association recommends 1.5 g (which equates to 65 mmol of sodium or 3.8 g salt) for high-risk patients, including those with CKD [71]. The 2021 KDIGO guidelines recommend targeting a sodium intake <2 g of sodium per day (or <90 mmol of sodium per day, or <5 g of sodium chloride per day) in patients with high blood pressure and CKD but warns that this recommendation is based on weak evidence (2C grade) [72]. An analysis of the Chronic Renal Insufficiency Cohort (CRIC) showed a mean sodium intake of about 3720 mg/day, indicating that the vast majority of CKD patients in this cohort, representative of the CKD population in the USA, do not comply with this recommendation [73].

**Figure 2:**
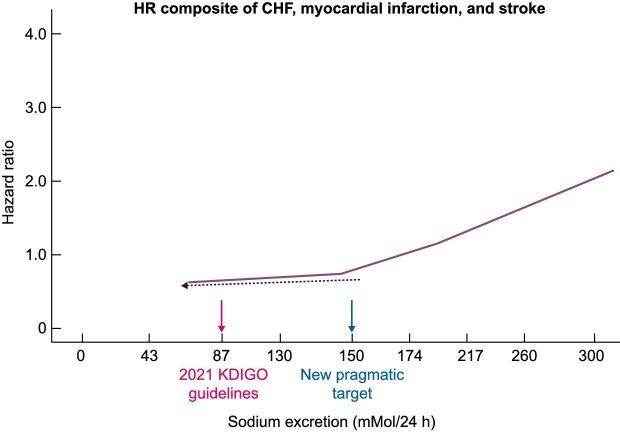
Figure redrawn from Mills *et al*. [73] (*JAMA* 2016;315:2200–10) showing the important relationship between sodium excretion and risk of major cardiovascular events. Below 150 mMol Na excretion/24 h, the risk for major cardiovascular events remains almost constant.

Long-term studies suggest low sodium intake contributes to retard kidney disease progression in diabetic patients treated with an angiotensin receptor blocker. This was nicely demonstrated in the ‘Irbesartan in Diabetic Nephropathy Trial’ (IDNT) and the ‘Reduction of Endpoints in Non-insulin-dependent diabetes mellitus with the Angiotensin II Antagonist Losartan’ (RENAAL) trial [74]. On the other hand, an analysis of the ‘Ramipril Efficacy In Nephropathy’ (REIN) study in nondiabetic CKD patients showed that an increase in sodium intake of 100 mmol/g creatinine imposes a 61% risk excess for kidney failure [75]. A meta-analysis by Garofalo *et al*. in 2018 retrieved 11 trials, including 738 stage G1–4 CKD patients testing low salt diets vs various comparator diets [76]. Urinary sodium excretion, the best surrogate of sodium intake, was 104 mEq/day and 179 mEq/day in low- and high-sodium intake subgroups, respectively, with a mean difference of −80 mMol/day. Overall, mean differences in the clinic and ambulatory systolic blood pressure were −4.9 mmHg and −5.9 mmHg, respectively. The mean difference in proteinuria between the two diets was −0.39 g/day. No major clinical outcome (progression to kidney failure, cardiovascular events) was reported in these trials that were of short or very short duration. Indeed, three of these trials lasted only 2 weeks or less, and only two were 24 months long. Thus, globally, a diet containing about 100 mMol sodium/day produces a clinically relevant reduction in systolic blood pressure (about 5 mmHg) and proteinuria (a reduction of about 0.4 g/day) compared with diets with an average sodium content of about 180 mMol/day. An important finding that emerged in this meta-analysis was that the greater the duration of the salt intervention (>4 vs <4 weeks), the lower the achievement of the target salt restriction, pointing to difficulties in implementing salt restriction recommendations in CKD patients. The long-term sustainability of a low-salt diet is the major barrier to long-term dietary salt reduction. The high sodium content of processed foods coupled with the scarce attention given to reading the labels of processed food by patients, and more in general, the low awareness of the health risks of a high salt intake, concur to lower adherence to low salt diets. Complex educational interventions, including self-measurement of urinary sodium [77] or urinary chloride [78], had only modest and transient effects on adherence and small effects on BP. It is important to emphasize that the average sodium intake achieved in the trials included in the meta-analysis discussed above was about 100 mMol/day, i.e. 2.3 g of sodium and 5.8 g of salt. In the highly controlled scenario of clinical trials, only half of CKD patients achieve the target sodium intake in the general population (2.3 g) [70] and even fewer the 2.0 g target recommended by the 2021 KDIGO guidelines [72]. In clinical practice and in busy renal units, an even worse adherence to dietary recommendations is likely. Globally, the evidence supporting using a low-salt diet in CKD patients remains weak. An important longitudinal study in the CRIC cohort that accurately measured sodium intake by applying at least three 24 h urine collections clearly demonstrated an almost linear relationship between sodium intake and the risk of cardiovascular events [73]. A close examination of this relationship shows that, below the 150 mMol/24 h threshold, the risk reduction for these events is very small (Fig. 2).

Box 5: The long-term sustainability and adherence to a low-salt diet is a major barrier to long-term dietary salt reduction. The high sodium content of processed foods coupled with the scarce attention given to reading the labels of processed food by patients, and general low awareness of the health risks associated with a high salt intake, concur with a low adherence to low salt diets.

On the other hand, we should not forget the value of diuretics in CKD. A randomized, 6-week crossover trial in 26 stage 3–4 CKD patients testing a hydrochlorothiazide/amiloride combination vs a 60 mMol/day sodium diet showed that the diuretic combination was non-inferior to dietary sodium restriction in reducing blood pressure and extracellular volume [79]. Furthermore, chlorthalidone's long-term effectiveness and safety for controlling resistant hypertension were more recently confirmed in the ‘Chlorthalidone for Hypertension in Advanced Chronic Kidney Disease’ trial [80]. Thus, a pragmatic sodium target of about 150 mMol/day (about 9 g salt) coupled with judicious use of diuretics appears a rational approach for limiting sodium and volume excess in CKD patients. However, the strategy remains to be tested in clinical trials of CKD patients evaluating clinical endpoints.

Box 6: Diuretics were shown to be non-inferior to dietary sodium restriction in reducing blood pressure and extracellular volume.

A more pragmatic sodium target of about 150 mM per day coupled with judicious use of diuretics may be a more rational approach in limiting sodium and volume excess in CKD patients but warrants further evaluation in a randomized trial setting.

## CONCLUSIONS

The scientific basis of a restricted protein diet and a restricted salt diet in CKD has been recognized for at least half a decade. However, high quality clinical evidence to guide recommendations and thresholds level for dietary protein and salt intake in CKD patients are still lacking. A key barrier to building quality evidence examining hard outcomes in this area as well as patients’ acceptance and long-term adherence to a restricted diet prescription in CKD patients relates to practical difficulties in achieving sustainability of a ‘restricted’ salt and ‘restricted’ protein diet, and practical difficulties in adopting the same restricted diets across different patients population and racial groups. Many patients find the restricted diet unpalatable. Being able to enjoy good food without restrictions is an important aspect that impacts patients’ quality of life and needs to be taken into consideration in the delivery of kidney nutrition care. Many patients also do not recognize the hidden salt, phosphate and other additives in processed foods, and seldom read food labels. Kidney nutrition care thus requires a highly personalized approach involving shared decision-making with patients/carers, taking into consideration patients’ wishes, lifestyle, life goals, pre-existing dietary habits, health literacy, incentives and motivations, as well as CKD stages and other pre-existing comorbidities. Patients and carers’ education are crucial to bridge the knowledge gaps many patients and carers may have as to what constitutes a ‘healthy kidney diet’. Patients should be promoted and engaged in self-management tools of dietary intervention, for example utilizing mobile technologies. With the advent of novel drug therapies for CKD management, studies will be needed to evaluate the incremental role of a restricted protein and a restricted salt diet in improving clinical outcomes and patient-centred outcomes in CKD patients.

## Data Availability

No new data were generated or analysed in support of this research.
